# Reverse Engineering Provides Insights on the Evolution of Subgroups A to E Avian Sarcoma and Leukosis Virus Receptor Specificity †

**DOI:** 10.3390/v11060497

**Published:** 2019-05-30

**Authors:** Mark J. Federspiel

**Affiliations:** Department of Molecular Medicine, Mayo Clinic, Rochester, MN 55905, USA; federspiel.mark@mayo.edu

**Keywords:** Avian Sarcoma and Leukosis Viruses, receptor usage, envelope glycoprotein evolution

## Abstract

The initial step of retrovirus entry—the interaction between the virus envelope glycoprotein trimer and a cellular receptor—is complex, involving multiple, noncontiguous determinants in both proteins that specify receptor choice, binding affinity and the ability to trigger conformational changes in the viral glycoproteins. Despite the complexity of this interaction, retroviruses have the ability to evolve the structure of their envelope glycoproteins to use a different cellular protein as receptors. The highly homologous subgroup A to E Avian Sarcoma and Leukosis Virus (ASLV) glycoproteins belong to the group of class 1 viral fusion proteins with a two-step triggering mechanism that allows experimental access to intermediate structures during the fusion process. We and others have taken advantage of replication-competent ASLVs and exploited genetic selection strategies to force the ASLVs to naturally evolve and acquire envelope glycoprotein mutations to escape the pressure on virus entry and still yield a functional replicating virus. This approach allows for the simultaneous selection of multiple mutations in multiple functional domains of the envelope glycoprotein that may be required to yield a functional virus. Here, we review the ASLV family and experimental system and the reverse engineering approaches used to understand the evolution of ASLV receptor usage.

## 1. Introduction

Enveloped viruses continue to be major pathogens for humans and animals, but are also being harnessed as therapeutic tools for medical therapies. Whether pathogenic or therapeutic, the initial infection and subsequent dissemination of the virus depends on efficient entry into susceptible host cells. A detailed understanding of the mechanisms of viral entry may provide new targets for antiviral therapies to combat viruses evolving to cross species boundaries and expanding virulence.

To enter cells and begin replication, enveloped viruses must fuse the membrane coating the viral particle with a cellular membrane to deliver a subviral particle inside the cell. Enveloped viruses use one or more viral encoded glycoproteins to mediate the fusion of the viral and host cell membranes. This is a thermodynamically favored process but with one or more very high energy barriers. The energy liberated upon conformational changes in the viral glycoproteins is used to overcome the energy barrier(s) (reviewed in [1,2,3,4,5]). Several viral fusion glycoproteins have been studied in great detail with structures of the initial viral glycoprotein before the fusion process begins (pre-fusion), and a postfusion viral glycoprotein structure after all conformational changes have occurred. While the viral glycoproteins have very different sequences and molecular architectures, the similarity of the postfusion structures suggests that they all mediate fusion by a similar mechanism.

**Figure 1:**
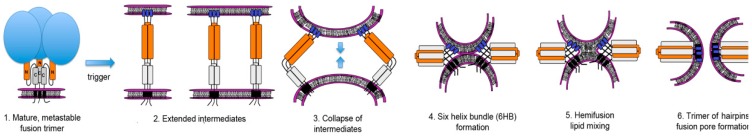
Current model of Class 1 viral fusion protein mediated membrane fusion pathway.

A general outline of the fusion process mediated by enveloped virus fusion proteins as currently proposed is shown in Figure 1 for class I viral fusion proteins as an example.

(1) Many of the viral fusion glycoproteins are trimers of heterodimers. Each heterodimer consists of a globular, receptor binding domain (blue spheres) and a fusion protein that consists of a fusion peptide, two regions of tertiary structure (N, C), and a region to anchor the heterodimer in the viral membrane. The trimer of dimers usually undergoes a late protease cleavage resulting in a trapped, metastable, fusion-active conformation that requires a trigger to begin the fusion process.
(2) After interaction with the appropriate trigger, the fusion-active glycoprotein undergoes an extensive conformation change producing an extended intermediate form that delivers the hydrophobic fusion peptide (blue box) to bury in the target cellular membrane, linking the viral and cellular membranes. Multiple extended viral glycoprotein intermediates are thought to be necessary to form a fusion pore. The globular domains are not shown for clarity.
(3) The extended intermediates are energetically favored to collapse.
(4) This collapse forms the stable six-helix bundle (6HB) and draws the two membranes together.
(5) The apposition of the two membranes causes disruption and mixing of the lipid leaflets, a state called hemifusion.
(6) A fusion pore is the result of the final conformational changes in the viral glycoproteins forming a trimer of hairpins. Once triggered, the conformational changes in the viral fusion proteins are thought to occur in a relatively short time resulting in the trimer of hairpins, the thermodynamically favored final structure.

## 2. Classes of Viral Fusion Proteins

While all of the viral fusion proteins have a similar overall organization being type I integral membrane proteins that form trimeric structures as their fusion-active form, and form a trimer of hairpins structure postfusion, there are some differences especially in the secondary structures adopted by the fusion intermediates and final postfusion forms (reviewed in [1,2,3,4,5]). Class I fusion proteins (e.g., Influenza virus HA and Retroviruses Env glycoproteins) have alpha-helical secondary structures that form (Figure 1: N, C) the extended intermediate, 6HB, and trimer of hairpin structures. Class I fusion proteins have a trimer structure in the native protein, are proteolytically cleaved to form a metastable fusion-active state, and are displayed as spikes, perpendicular to the membrane surface. Class II fusion proteins (e.g., TBEV E) use beta-sheet secondary structures to form the extended fusion intermediate and final postfusion structures. However, the native structure of the fusion protein is a dimer that lies parallel to the membrane. As with Class I proteins, Class II fusion proteins require proteolytic cleavage to form the fusion-active state. Recently, a third class of viral fusion proteins has been identified. Class III viral fusion proteins (e.g., VSV G) have both alpha-helical and beta-sheet secondary structures in their extended fusion intermediates and final postfusion forms. These fusion proteins do not require proteolytic processing to form an active fusion state, nor are the proteins in a metastable form on the virion surface. In addition, while the fusion peptides of Class I and II fusion proteins are buried in the subunit interfaces of the native protein, the fusion peptide region of Class III proteins are exposed. Despite the different structural forms of the N-terminal and C-terminal regions of the fusion proteins, as well as other differences in presentation on the virion surface, the fusion proteins of all three classes are proposed to follow the fusion mechanism depicted in Figure 1.

## 3. Viral Fusion Protein Triggers

As mentioned above, most viral fusion proteins require oligomerization and at least one proteolytic cleavage after synthesis to produce a fusion-active protein complex. The subsequent conformation changes needed for fusion are then locked in a metastable form until an appropriate trigger releases the lock. Up until recently, the triggering viral fusion protein conformational changes was thought to occur by two mechanisms. One mechanism uses low pH exposure in an endosomal compartment to induce the conformational changes leading to the extended intermediate form exposing the fusion peptide to the target membrane (Figure 1: step 1 to 2). Influenza virus is a well-studied example of this mechanism where the HA1 subunit binds to sialic acid causing the trafficking of the virion to the endosomal compartment, but only low pH exposure causes conformational changes in the fusion protein. In another triggering mechanism, the interaction of the viral fusion protein with a specific cellular receptor protein enables the conformational changes to occur at a neutral pH at the cell surface. Most retroviruses are thought to use this mechanism. However, HIV-1, a complex retrovirus, has broken this neutral pH process into two steps, using first CD4 to trigger an initial but partial conformational change in the envelope glycoproteins that enables the binding of a second receptor, CCR5, to complete the triggering process. More recently, a third mechanism has been described for triggering conformational changes in viral fusion proteins: a two-step process that combines receptor triggered conformational changes at neutral pH followed by a required low pH exposure to complete the fusion process. The Avian Sarcoma and Leukosis Virus (ASLV) subgroup A (ASLV(A)) envelope glycoproteins are the most studied example of this two-step mechanism [6,7,8].

## 4. ASLV Experimental System

The ASLV family has members that lack oncogenes, ALVs, and cause disease from chronic infection and integration often activating an oncogene to cause disease [9,10]. The ASV members cause an acute disease from carrying an activated oncogene: Rous sarcoma virus (RSV) containing the oncogenic *src* is a well-studied example. In cultured avian cells, infection by an RSV will result in an obvious morphology change as a result of transformation by *src* of the infected cells that was developed into an infectious titer assay. However, ALVs often can infect and spread in a culture without obvious morphology changes. The complete family of ASLVs has recently been divided into 11 subgroups, A through K, based on their envelope glycoproteins and receptor usage patterns in susceptible and resistant avian cells, with subgroups A to E ASLVs being the most studied [11,12,13,14,15]. These ALVs have been classified into noncytopathic (subgroups A, C, and E) and cytopathic (subgroups B and D) viruses depending on whether they induced cytotoxicity in cultures avian cells. The ASLV induced cytotoxicity is not fusion of multiple cells to form syncytia, but rather a slowing of cell replication with the rounding and release of dead cells from the matrix. However, we have observed some subgroup C strains causing cytotoxicity in certain avian cells with the length and severity of the cytotoxicity appearing to be correlated with the expression levels of the viral glycoproteins.

### 4.1. ASLV Subgroup A to E Envelope Glycoproteins

The ASLV subgroup A through E (ASLV(A) through ASLV(E)) are a group of highly related alpharetroviruses that have evolved their *env* genes, which encode the viral envelope glycoproteins, from a common ancestor to use members of very different host protein families as receptors to enable efficient virus entry [16,17]. The evolution to use alternative receptors was presumably due to the development of host resistance and/or to expand host range. As with all retroviruses, ASLV viruses initially synthesize their envelope glycoproteins as a precursor polyprotein that forms a trimer. The final maturation step cleaves each polyprotein precursor of the trimer into two glycoproteins: the surface glycoprotein (SU), which contains the major domains that interact with the host receptor, and the transmembrane glycoprotein (TM) that anchors SU to the membrane with a stable, covalent disulfide bond [8,18], and is directly involved in the fusion of the viral and host membranes. This cleavage results in the mature, metastable, fusion-active complex, a trimer of SU:TM heterodimers.

**Figure 2:**
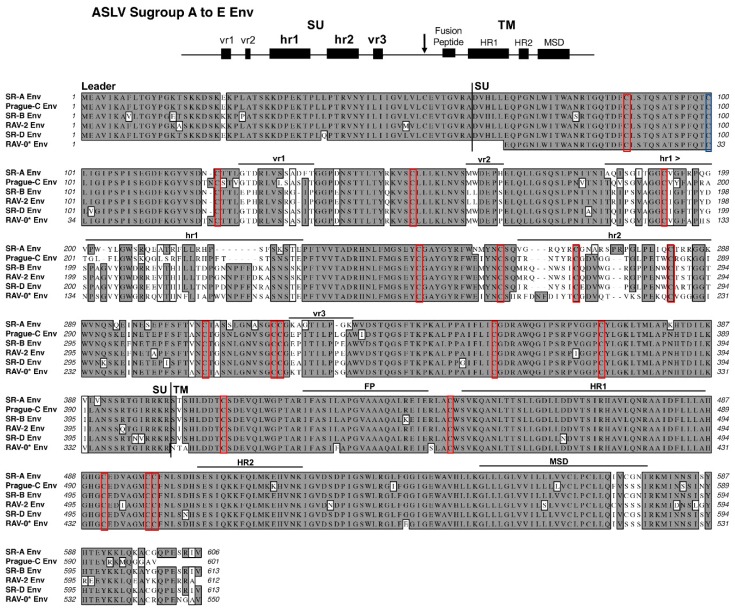
Schematic representations of the major functional domains and comparison of representative Avian Sarcoma and Leukosis Virus (ASLV) subgroups A to E envelope glycoprotein sequences. The envelope glycoprotein leader sequence (Leader), surface glycoprotein sequence (SU), transmembrane glycoprotein sequence (TM) are indicated. The variable region (vr1, vr2, and vr3) and the host range region (hr1 and hr2) sequences in the surface glycoprotein, and the fusion peptide (FP), heptad repeat (HR1 and HR2), and the membrane spanning domain (MSD) sequences in the transmembrane glycoprotein are indicated. The cysteine residues are highlighted in red boxes; the one unpaired cysteine residue at position 100 is highlighted with a blue box. The sequence alignments were done using the ClustalW program in MacVector 14.5.3: identical residues are shaded; conserved residue differences are in boxes, and nonconserved residue differences are unmarked. SR-A: Schmidt–Ruppin A subgroup A ASLV strain UniProt P03397; SR-B: Schmidt–Ruppin B subgroup B ASLV Genbank AAC08989; RAV-2, this study and Genbank AAA87241; Prague-C subgroup C ASLV Genbank AAB59934.1; SR-D: Schmidt–Ruppin D subgroup D ASLV Genbank BAD98245.1; RAV-0* is a partial sequence of a subgroup E ASLV and is the combination of two partial sequences: Genbank AAA87242 and CAA30677.

The ASLV(A) through ASLV(E) SU glycoproteins are highly conserved except for five variable domains, vr1, vr2, hr1, hr2, and vr3 (Figure 2). A variety of studies have identified hr1 and hr2 as the principle binding domains between the viral glycoprotein trimer and the host protein receptor, with vr3 contributing to the specificity of the receptor interaction for initiating efficient infection [19,20,21,22,23,24,25]. The ASLV TM glycoproteins contain an internal fusion peptide (FP), thought to project toward the host cell membrane upon the triggering of the metastable structure, and two domains in, the N-terminal heptad repeat (HR1) and the C-terminal heptad repeat (HR2), are critical for the formation of the extended structure and subsequent formation of the trimer of hairpins fusion structure. Finally, the membrane spanning domain (MSD) anchors the TM glycoprotein into the membrane.

**Figure 3:**
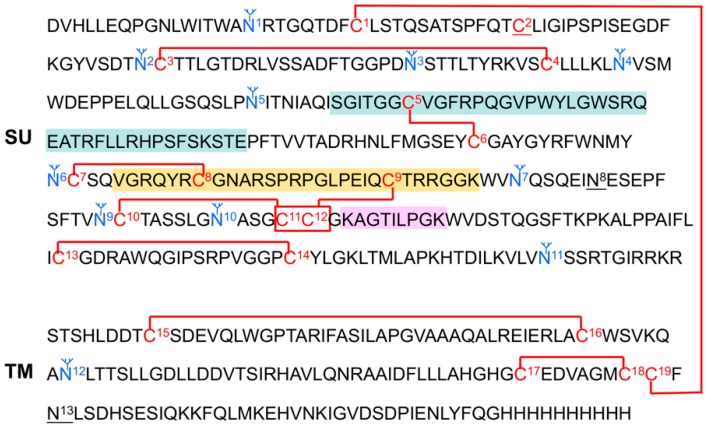
The proposed secondary structure of the subgroup A to E ASLV envelope glycoproteins. Shown are the disulfide bonds determined using a His-tagged, secreted form of the RCASBP(A) envelope glycoprotein expressed using chicken DF-1 cells and purified. The bonds to C11 and C12 (indicated with a box) could not be assigned further. The free cysteine is labeled C2. The N-linked glycosylation sites actually containing carbohydrate are marked in blue. The unglycosylated sites are underlined, N8 and N13. For reference, the hypervariable regions are marked: hr1 in green boxes; hr2 in an orange box; and vr3 in a pink box.

Disulfide bond exchange has been shown to be a critical step in the triggering and/or fusion process of some class 1 viral fusion proteins [26,27,28,29,30,31,32,33,34,35]. For viruses that have a CXXC thiol-disulfide exchange motif, for example HTLV-1 and MLV SU glycoproteins, the free Cys of this motif reduces the intersubunit disulfide bond linking the SU and TM glycoproteins: this is an integral step for the fusion process. Other viruses do not have the CXXC motif, for example ASLV Env and Ebola virus GP, and at least some of these viruses appear to retain the intersubunit disulfide bond throughout the fusion process, for example ASLV Env [8,18]. A recent report by Smith and Cunningham studying the multistep fusion and entry mechanism of ASLV(A) identified at least one Cys residue that formed a reactive Cys-thiolate upon receptor-triggered conformational changes that was required for functional fusion and infection [18]. This Cys-thiolate did not mediate isomerization of the SU-TM disulfide bond as expected: the precise function of this thiolate is still unclear. In addition, the authors present evidence that at least two other Cys residues that formed thiolates after receptor triggering on Env. However, these residues could not be studied further since the mutant protein could not fold. In our recently published study (see below) we mapped the disulfide bond pattern of the pre-fusion form of ASLV(A) Env [36]. There are 19 Cys residues in ASLV(A) Env; one residue must be free (Figure 3). The second Cys residue was found to be free. Interestingly, the same Cys residue that forms the thiolate required for fusion and infection. Since all 19 cysteine residues are conserved between subgroup A to E envelope glycoproteins, we are assuming that the disulfides bonds determined using a subgroup A glycoprotein are also conserved for the other subgroups.

### 4.2. ASLV Subgroup A to E Receptors

**Figure 4:**
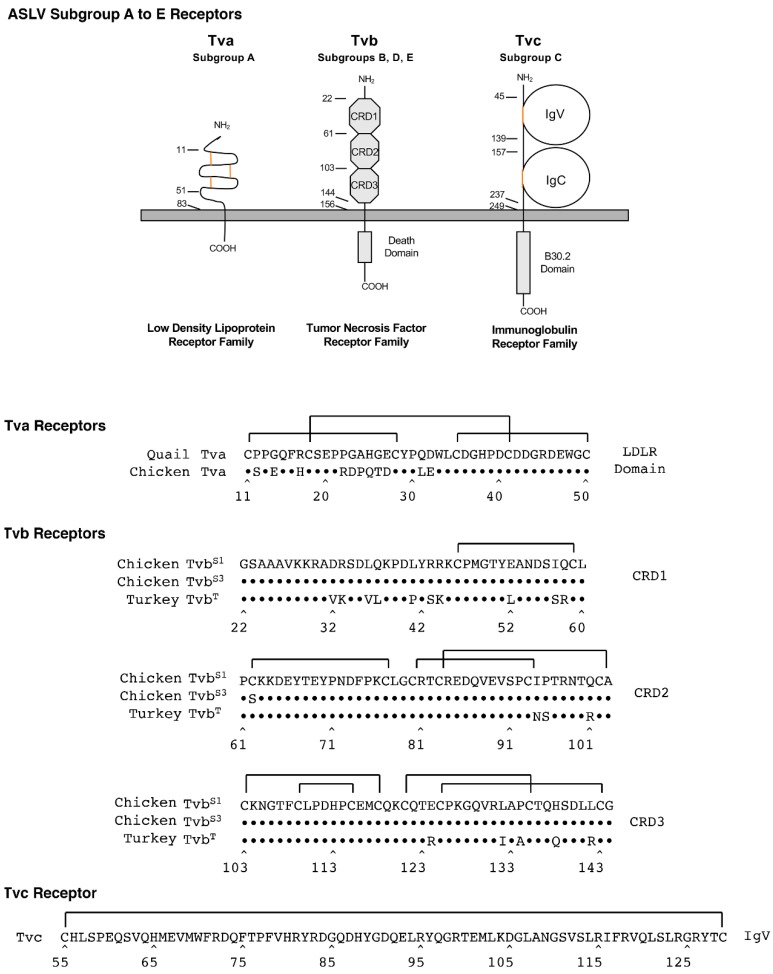
Schematic representations and protein sequences of critical domains of the three families of cellular surface proteins used as subgroup A to E ASLV receptors.

Members of three very different families of proteins have been identified to be receptors of these five ASLVs; all are simple, single-spanning membrane proteins (Figure 4).

Tva proteins are related to low-density lipoprotein receptors (LDLR) and are receptors for ASLV(A) [37,38]. LDLRs usually contain multiple cysteine-rich regions; Tva has one cysteine-rich LDLR region located between residues 11 and 50 in the extracellular domain with three required disulfide bonds between the six cysteine residues. Initial studies identified the carboxyl-terminal half of this 40-amino acid region was required for ASLV(A) receptor function. Three residues in the protein loop formed by the C4–C6 disulfide bond, Asp46, Glu47 and Trp48, as well as several other residues in the carboxy-terminal half of the LDLR domain were identified as critical for the efficient interaction of the quail Tva receptor and the ASLV(A) glycoproteins in a variety of assays [39,40,41,42,43,44]. It was initially thought that since the chicken Tva receptor was identical to quail Tva in this carboxy-terminal half, that chicken Tva interactions with ASLV(A) glycoproteins would be similar. However, a genetic evolution experimental system selected ASLV(A) variants with mutations in the envelope glycoproteins that could now preferentially use the chicken Tva receptor with high binding affinity and infection efficiency but not the quail Tva homolog [22,23,45]. Several critical residues in the amino-terminal half of Tva were found to account for this preference: residues at positions 11, 14, and 31 [46].

Tvb proteins are related to tumor necrosis factor receptors (TNFR) and are receptors for ASLV(B), ASLV(D), and ASLV(E) [47,48,49,50,51]. TNFR-related proteins contain three cysteine-rich domains (CRDs) in the extracellular domain. The chicken Tvb^S1^ is a receptor for subgroups B, D, and E; the chicken Tvb^S3^ is a receptor for subgroups B and D (10); and the quail Tvb^Q^ and the turkey Tvb^T^ are receptors for only subgroup E. Originally, the major determinants of the ASLV(B), ASLV(D) and ASLV(E) glycoproteins with the Tvb receptors were thought to be independent of the CRD3 region. 

A 15-residue region in CRD1, extending from residues 32 to 46, was reported to contain the critical determinants for efficient interaction with subgroups B and D ASLVs. Specifically, residues L36, Q37, L41, and Y42 were critical for high binding affinity and efficient entry, and the disulfide bond was not required. Subgroup E ASLVs, in contrast, required Tvb residues in both CRD1 and CRD2 for efficient virus binding and entry, including intact disulfide bonds and residues Y67, N72, and D73. The Tvb^S3^ protein contains the C62S substitution, which presumably alters the structure of the CRD2 domain, eliminating binding of ASLV(E) but having no effect on ASLV(B) and ASLV(D) binding and virus entry. More recently, the Cys125S substitution in CRD3 of Tvb^S1^ was shown to significantly reduce the binding affinity of the mutant receptor for all three ASLV subgroups, B, D, and E. This was the first demonstration of a possible role of CRD3 in Tvb function as an ASLV receptor [52].

Tvc proteins are related to mammalian butyrophilins and are members of the immunoglobulin protein family, and are receptors for ASLV(C) [53,54]. The extracellular domain of Tvc contains two immunoglobulin-like domains, IgV and IgC, which presumably each contain a disulfide bond important for native function of the protein. All of the functional determinants of Tvc function are contained in the IgV domain [55]. Residues Trp48 andTyr105 were identified as critical for high binding affinity interaction with the subgroup C ASLV glycoproteins and efficient infection. However, while the specific IgC domain was not required, additional experiments demonstrated that an additional domain was necessary as a spacer between the IgV domain and the membrane-spanning domain for efficient Tvc receptor activity, most likely to properly orient the IgV domain in a proper distance from the cell membrane.

The ASLV receptor alleles have been studied in a variety of susceptible and resistant strains of chicken [49,52,54,56,57,58]. A variety of mutations were identified that either result in a severely truncated or complete absence of the receptor protein, or point mutations often changing cysteine residues and altering receptor protein folding to reduce the binding affinity between the mutant receptor protein and the ASLV Env trimer (Table 1). These studies provide some examples of the natural receptor variants encountered by ASLVs that may have led to the evolution of receptor usage and the evolution of the subgroup A-E ASLVs. Recently, several chicken cell lines were engineered using CRISPR/Cas9 technology to induce genetic resistance by inserting deletions into receptor genes that knock out expression, e.g., Tva and Tvc [59]. New experimental models can now be engineered using this type of technology to ask additional questions.

### 4.3. ASLV Receptor and Glycoprotein Immunoadhesins

**Figure 5:**
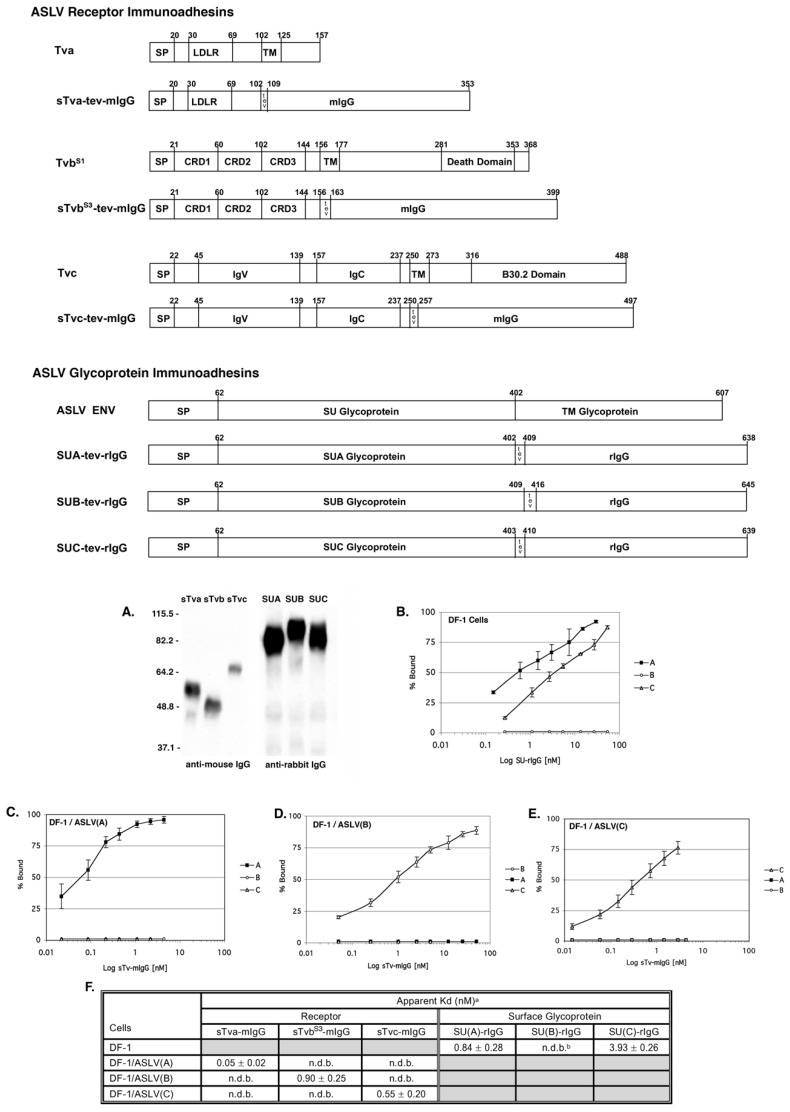
Schematic representations and binding affinities of ASLV receptor and glycoprotein immunoadhesins. (**A**) Western immunoblot analysis of the soluble forms of the chicken Tva receptor, sTva-mIgG (sTva), the Tvb^S1^ receptor, sTvb^S1^-mIgG (sTvb) and the Tvc receptor, sTvc-mIgG (sTvc), immunoprecipitated with anti-mouse IgG-agarose beads, and the secreted forms of the SU glycoproteins SU(C)-rIgG (SUC), SU(A)-rIgG (SUA), and SU(B)-rIgG (SUB) immunoprecipitated with anti-rabbit IgG-agarose beads. The precipitated proteins were denatured, separated by SDS-12% PAGE, and transferred to nitrocellulose. The filters were probed with either peroxidase-conjugated goat anti-mouse IgG or goat anti-rabbit IgG, and the bound protein–antibody complexes visualized by chemiluminescence using Kodak X-Omat film. Molecular sizes (in kilodaltons) are given on the left. (**B**–**E**) Uninfected DF-1 cells (**B**) and DF-1 cells chronically infected with either ASLV(A) (**C**), ASLV(B) (**D**), or ASLV(C) (**E**) and uninfected DF-1 cells (**B**) were fixed with paraformaldehyde and incubated with different amounts of each secreted SU-rIgG (**B**) or each soluble receptor–mIgG (C–E). The receptor–viral glycoprotein complexes were bound to either goat anti-mouse IgG or goat anti-rabbit IgG linked to phycoerythrin. The amount of phycoerythrin bound to the cells was measured by FACS, and the maximum fluorescence was estimated. The data were plotted as percent maximum fluorescence bound versus concentration of the soluble receptor–mIgG or secreted SU-rIgG. The values shown are averages and standard deviations (error bars) of three experiments. (**B**) A, SU(A)-rIgG; B, SU(B)-rIgG; C, SU(C)-rIgG. (**C**–**E**): A, sTva-mIgG; B, sTvb^S1^-mIgG; C, sTvc-mIgG. (**F**) Estimated binding affinities of the soluble forms of the ASLV receptors for ASLV envelope glycoproteins expressed on the surface of infected DF-1 cells, and soluble forms of the ASLV surface glycoproteins for endogenous levels of the ASLV receptors expressed on DF-1 cells. ^a^ Apparent Kd values were estimated by fitting the data via nonlinear least squares to a log logistic growth curve function as described in Materials and Methods. Each result is the average and standard deviation from three experiments. ^b^ n.d.b.—no detectable binding. Gray fields indicate binding reactions not performed.

We, and others, have shown that the ASLV SU glycoprotein and the extracellular region of the ASLV receptors contain the necessary regions that interact to determine subgroup specificity and for high affinity binding. Expressing the SU and receptor extracellular domains fused to a region of an IgG, immunoadhesins, resulted in the production of soluble, secreted biologically active proteins with high stability from host chicken cells, the DF-1 fibroblast cell line (Figure 5). The IgG domain also enabled the use of a wide range of standard detection reagents, and well-established IgG purification protocols could be adapted for immunadhesin purification. For our studies we have consistently fused SU to a rabbit IgG (rIgG) and fused the extracellular domain of ASLV receptors to a mouse IgG (mIgG). In this way, a functional assay for SU:receptor interactions was developed that could coimmunoprecipitate the interacting proteins with either an anti-mIgG or an anti-rIgG sera. We have approached the definition of ASLV entry mechanisms by keeping as close to physiological conditions for ASLV replication as possible in vitro: avian cells in which the receptors, viral proteins, and secreted immunoadhesins are synthesized and post-translationally modified as the wild type proteins, and expressed on the cell surface at wild type levels. The ASLV SU and receptor immunoadhesins have provided valuable tools for studying the mechanisms of retroviral entry including the identification of functional interaction determinants of SU and receptor for efficient virus entry [22,23,45,53,57,60]. Finally, the receptor–mIgG immunoadhesins can trigger conformational changes in the mature ASLV Env trimers indistinguishable from a natural, membrane-bound receptor.

### 4.4. RCAS Family of Replication-Competent ASLV Vectors

The ASLV family of avian viruses naturally included examples of replication-competent isolates that contained the *gag*, *pol*, and *env* genes required for replication, but also Rous sarcoma virus (RSV) isolates that contained an additional oncogene *src*. The ability to efficiently replicate and carry an additional transgene was unusual and Stephen Hughes’s lab constructed the RCAS family of replication-competent vectors based on RSV (reviewed [61,62]). RCAS stands for Replication-Competent ALV LTR that contains an enhancer/promoter with a Spice acceptor in front of a unique *ClaI* site for inserting transgenes. The RCAS family of replication-competent viruses had enabled exquisitely detailed studies not only characterizing the replication process of this model retrovirus, but also enabling the evolution of new, variant viruses capable of evading a specific block to replication and thereby identify functional residues.

Historically, the RCAS vectors were constructed using the SR-A virus and consequently have subgroup A ASLV envelope glycoproteins, RCAS(A). It was known at the time that the SU glycoprotein hypervariable region was sufficient to determine receptor specificity. In addition, the C-terminal region of SU glycoprotein and the extracellular region of the TM glycoprotein were nearly identical across the subgroup A-E ASLVs (Figure 2). Therefore, RCAS vectors with other envelope glycoprotein subgroups were then constructed by only replacing the *env* gene segment containing the RCAS(A) SU hypervariable regions with the corresponding segment of another ASLV subgroup. For example, the subgroup B SU hypervariable region from RAV-2 was used to create RCAS(B), while the same SU region of subgroup C Prague C RSV was used to create RCAS(C), but the remaining glycoprotein sequence is from the SR-A isolate.

However, while the C-terminal SU and TM glycoprotein regions are nearly identical, recently, we and others have identified several differences in the C-terminal SU and TM regions that can be important for efficient ASLV entry. Therefore, the sources of the actual regions of a specific RCAS envelope glycoprotein are critical for an accurate evaluation and understanding of any ASLV entry experiment.

The subgroup A RCASBP vectors replicate to the highest titers, >10^6^ ifu/mL in DF-1 cells, possibly due to not inducing any cytotoxicity. Subgroups B and C RCASBP vectors replicate to lower titers, ~10^5^ ifu/mL, in DF-1 cells likely due to the cytotoxic effect of their glycoproteins on the cells.

### 4.5. Receptor Usage Assays

**Figure 6:**
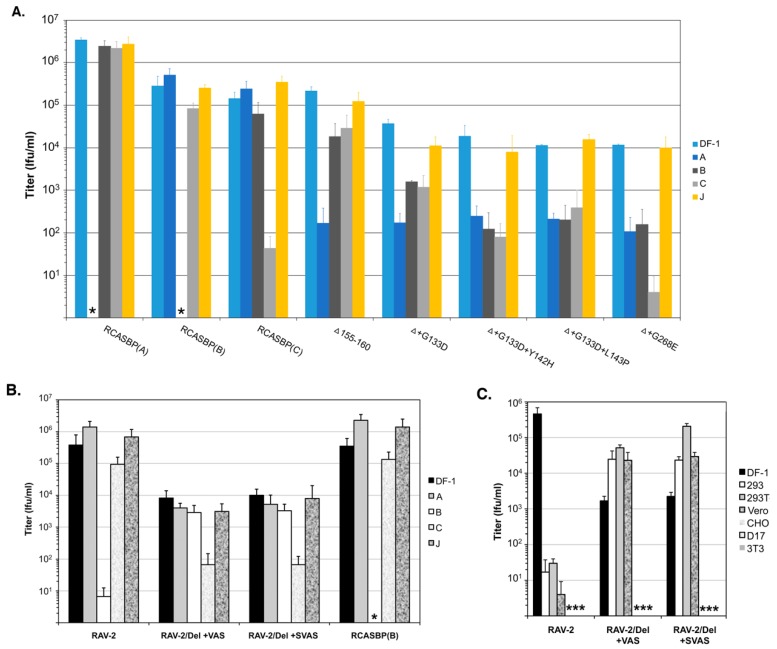
Examples of analyses of receptor usage of the wild type and mutant ASLVs. Infectious titers were determined using 10-fold serial dilutions of wild type RCASBP(A), RCASBP(B), and RCASBP(C) viruses, the parental Δ155–160 virus, and the Δ155–160 mutant virus supernatants produced using DF-1 cells. The infectious titer was determined by the AP assay. No infectious units detected are denoted with (∗). The results shown are an average of three different experiments; error bars show standard deviations. (**A**) ASLV receptor interference patterns of the ASLVs infecting parental DF-1 cells, and DF-1 cells chronically infected with ASLV(A), ASLV(B), ASLV(C), or subgroup J ASLV, HPRS103. The replicative abilities and receptor usage of RAV-2/Del136–142 mutants. (**B**,**C**) The abilities of the ASLV parental viruses and mutants to alter receptor usage in avian cells using a receptor interference assay (**B**) using virus supernatants produced from transfected DF-1 cells (see above) titered on parental DF-1 cells (DF-1) and DF-1 cells previously infected by ASLV(A), ASLV(B), ASLV(C) or ASLV(J). The same viral supernatants were also assayed for their abilities to infect mammalian cells (**C**) that do not express ASLV receptors.

One powerful method used to organize the receptor usage of the ASLV family of viruses exploited the concept of receptor interference [13,14,15]. A key observation was cells previously infected by a particular subgroup ASLV, could not then be reinfected by a virus with the same subgroup glycoproteins. The infection block occurred due to the cells expressing the viral glycoproteins which bound and blocked that specific receptor required for entry of viruses requiring the same receptor. The RCAS vectors can be used to chronically infect cultures of avian cells, and the same subgroup vector containing a reporter gene (e.g., AP and GFP) can be used to challenge the cells and quantitate the infectious titer. Two examples of this type of assay are shown in Figure 6A,B [63,64]. The receptor interference assay demonstrates the remarkable receptor specificity for using just one receptor attained by the wild type ASLV envelope glycoproteins with little or no cross-interference. The subgroup J ASLV was used as a control since ASLV(J) encodes very different SU glycoproteins with only 40% identity compared to subgroups A to E. ASLV(J) also uses a very different type of cell surface protein as a receptor, the multimembrane spanning chicken Na^+^/H^+^ exchanger type 1 [65], and does not interfere with subgroups A to E infection nor is cross-neutralized with antisera. Mammalian cells do not express receptors for efficient ASLV infection and therefore are resistant to ASLV infection unless engineered to express Tva, Tvb, or Tvc receptors that then allows infection. However, while the ASLV can infect and subsequently integrate its genome into mammalian cells expressing an ASLV receptor, there are multiple blocks in ASLV replication that prevent the production on new infectious virus. As shown in Figure 6C, variants of ASLV can be selected that not only alter the ASLV receptor interference pattern but extend the host range to enable infection mammalian cells.

## 5. Subgroup A to E ASLV Entry Mechanism

**Figure 7:**
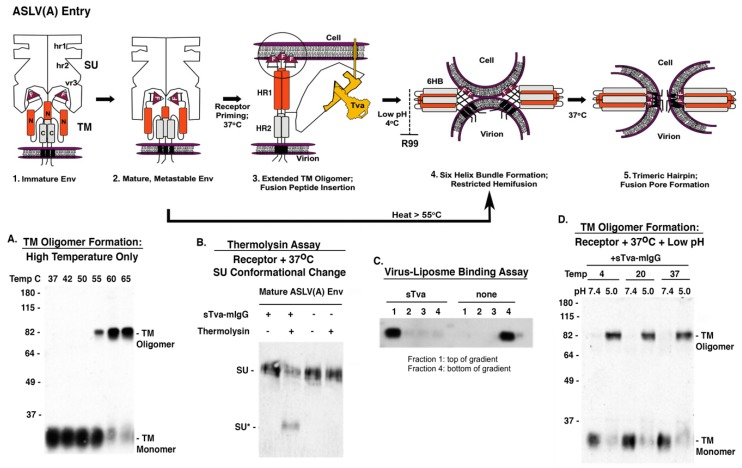
Current model of ASLV(A) Env mediated membrane fusion highlighting some of the stable intermediate stages that can be achieved experimentally with examples of biochemical assays that support the two-step fusion/entry mechanism of ASLV. For clarity, not all of the SU glycoproteins in the trimer are shown in steps 1–3; the SU glycoproteins are not shown in steps 4 and 5 but published reports indicate the disulfide bonds between SU-TM remain stable throughout the fusion process.

Subgroup A to E ASLV envelope glycoproteins belong to a group of class 1 viral fusion proteins with a two-step triggering mechanism that will allow experimental access to intermediate structures during the fusion process [6,7,8,66,67,68]. ASLV Env glycoproteins, as all retroviral glycoproteins, are initially synthesized as a polyprotein precursor with three precursor proteins oligomerizing to form a trimer. The final maturation step cleaves each polyprotein precursor of the trimer into the SU glycoprotein which contains the major domains that interact with the host receptor, and the TM glycoprotein that anchors SU to the membrane and is directly involved in the fusion of the viral and host membranes. ASLVs have an internal fusion peptide (F) flanked by Cys residues in a disulfide bond that forms a loop. This cleavage results in the mature, metastable, fusion-active complex (Figure 7, step 1 to 2). As with other mature viral fusion proteins, the mature ASLV Env glycoproteins are locked in a metastable conformation. Experimentally, the requirement for receptor triggering followed by low pH exposure to form the 6HB intermediate can be circumvented by treatment of the mature, metastable Env with a strong denaturant like high heat (>55 °C) (see Figure 7A).

ASLV Env glycoproteins have a stable, covalent disulfide bond linking the SU and TM subunits of each heterodimer: published data predicts the ASLV SU subunits to be bound to TM throughout the fusion process. Upon interaction of SU with an appropriate receptor protein, Tva for ASLV(A), Env undergoes conformational changes that result in the formation of an extended fusion intermediate that exposes the internal fusion peptide region for interaction with the target membrane (Figure 7, step 2 to 3). The triggering of the ASLV viral glycoprotein trimer upon receptor binding results in a conformational change in the SU glycoprotein that presumably separates the SU domains to allow the TM glycoproteins to form an extended structure projecting the internal fusion peptide (FP) toward the host cell membrane. Two domains in TM, HR1, the N-terminal heptad repeat (N, orange domain), and HR2, the C-terminal heptad repeat (C, grey domain), are critical for the formation of the extended structure. The fusion peptide is thought to interact with the target membrane irreversibly, forming an extended prehairpin TM oligomer structure anchored in both the viral and target membranes. The cooperation of several of these extended TM oligomers is thought to be necessary to complete the fusion process.

Physiological temperatures are required for receptor binding of SU to trigger a conformational change; receptor-SU binding at 4 °C does not induce a conformational change. The interaction of the ASLV(A) SU subunit with the Tva receptor protein at physiological temperatures triggers a conformation change in the SU glycoprotein revealing a novel cleavage site for thermolysin. The thermolysin assay uses a soluble form of the ASLV(A) receptor sTva-mIgG to trigger the SU conformational changes, followed by digestion with thermolysin, and the digestion products visualized by Western immunoblot using an anti-SUA monoclonal antibody revealing a smaller SU glycoprotein, SU* (Figure 7B). The receptor triggered conformation changes are also predicted to release the TM glycoprotein to form extended intermediates linking the viral and cellular membrane. A virus-liposome binding assay uses synthetic liposomes (~100 nm in size) as targets for TM fusion peptide binding creating stable intermediates with the liposomes that “float” higher in a sucrose density gradient demonstrating functional TM interactions with a target membrane (Figure 7C).

Presumably, this complex is then transported into an endocytic compartment and upon exposure to low pH, the ASLV extended or partially collapsed fusion intermediates fold into a six-helix bundle (6HB) conformation, bringing the viral and target membranes into close proximity. The viral and target membranes are brought into close proximity when the HR2 repeats (N, orange domains) fold back into the grooves formed by the HR1 repeats (C, grey domains), forming presumably the most stable TM structure, the six-helix bundle (6HB), and allowing the initial mixing of the outer lipid leaflets (hemifusion) (Figure 7, step 3 to 4). At 4 °C, only partial lipid mixing is possible (restricted hemifusion). The formation of the 6HB can be blocked by addition of the R99 peptide, a C-helix inhibitory peptide that binds to the N-helix blocking complete folding [69]. Upon low pH exposure, the receptor triggered ASLV glycoproteins fold back to form the stable SDS-resistant six helix bundle TM oligomer. TM oligomers in the 6HB form can be assayed using Western immunoblots with an anti-TM serum under defined SDS conditions demonstrating the requirement for physiological temperatures, first receptor triggering and followed by low pH exposure (Figure 7D). Addition of the inhibitory peptide R99 blocks the formation of the 6HB, thus the SDS-resistant TM oligomers are not detected.

The fusion of the membranes proceeds through a number of additional undefined steps and the 6HB may undergo additional structural rearrangements which requires physiologic temperatures to form the lowest energy trimer of hairpins structure, enabling the formation and expansion of the fusion pore and entry of the viral core into the cell to complete the fusion process (Figure 7, step 4 to 5).

So, the evolution of the ASLV envelope glycoproteins not only must evolve to specifically use a receptor with high binding affinity, but also maintain the ability to be productively triggered to allow specific initial structural conformations and then allow further structural conformations to mediate the efficient fusion of the viral and cellular membranes to complete entry. Interestingly, the Tva, Tvb, and Tvc receptors each contain one or more aromatic residues that are critical determinants for proper interaction with ASLV glycoproteins. This suggests that the ASLV glycoproteins may share a common mechanism of receptor interaction with an aromatic residue(s) on the receptor critical for proper triggering of the conformational changes in the glycoprotein trimer required for efficient virus entry.

## 6. Reverse Engineering: Identification of ASLV Envelope Glycoprotein Residues Critical for Virus Entry Using Genetic Selection Strategies

The initial step of retrovirus entry, the interaction between envelope glycoprotein trimer and a cellular receptor, is complex, involving multiple, noncontiguous determinants in both proteins that specify receptor choice, binding affinity, and the ability to trigger conformational changes in the viral glycoproteins. Despite the complexity of this interaction, retroviruses have the ability to evolve the structure of their envelope glycoproteins to use a different cellular protein as a receptor, often a protein that has no obvious homology to the original receptor, and retain efficient entry functions. Even using the highly homologous subgroup A to E ASLV glycoproteins, the complexity of a global mutational experimental approach is untenable. Therefore, we and others have taken advantage of the replication-competent ASLVs and exploited genetic selection strategies to force the ASLVs to naturally evolve and acquire envelope glycoprotein mutations to escape the pressure on virus entry and still yield a functional replicating virus. This approach allows for the simultaneous selection of multiple mutations in multiple functional domains of the envelope glycoprotein that may be required to yield a functional virus.

One genetic selection strategy has taken advantage of the fact that secreted forms of the ASLV receptors potently bind the Env trimer to compete with cell-associated receptors to block infection. The ASLV receptor immunoadhesins, the extracellular domain of an ASLV receptor fused to the mouse IgG domain (Figure 5), specifically bind only the concomitant ASLV glycoprotein subgroup to pressure the glycoprotein to acquire mutations that significantly reduce the binding affinity to the receptor immunoadhesin and possibly alter and/or broaden the ability of the variant to use other cell surface proteins as functional receptors.

A second strategy employs ASLV glycoprotein immunoadhesins, the SU glycoprotein domain fused to a rabbit IgG (Figure 5), to bind specifically to the concomitant receptor on the cell surface, to effectively reduce and/or eliminate its availability to bind incoming virus envelope glycoproteins and block infection. This approach applies evolutionary pressure to select ASLV glycoprotein variants with mutations that enable use an alternative receptor for virus entry.

A third genetic selection strategy has employed the wide variety of chicken lines that effectively do not have a functional ASLV subgroup receptor (Table 1 [56]), as well as other avian species that support ASLV replication but have limited or no receptor expression reducing or eliminating infection, which can then be used to select viral variants with glycoprotein mutations that expand receptor usage.

And finally, while the ASLVs do not replicate and produce new infectious virus in nonavian cells, nonavian cells can be engineered to express an ASLV receptor that allows infection, integration of the provirus, and some subsequent viral gene expression. Jan Svoboda and others have used mammalian cells to study the rare exceptions where RSVs were able to infect usually newly born rats or hamsters to express *src* and transform cells that were key to illuminate RSV biology. The ASLV experimental system has now been expanded with a variety of mammalian cell lines generated to express Tva, Tvb, or Tvc receptors and support that specific subgroup ASLV entry.

**Figure 8:**
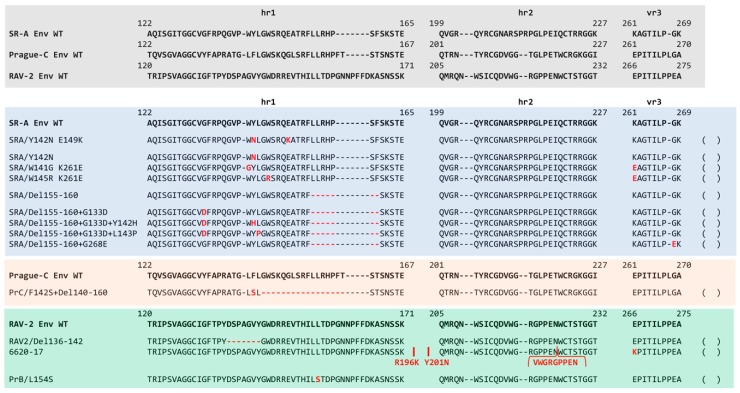
A summary and comparison of the ASLV SU glycoprotein wild type and escape mutations identified using various genetic-based selections strategies on limiting the entry and subsequent production of replication-competent ASLVs. Comparison of the SR-A, Prague C, and RAV-2 hr1, hr2, and vr3 hypervariable regions of the SU glycoproteins. The sequence alignments were done using the ClustalW program in MacVector 14.5.3: gaps are noted with black dashes (−). The identified mutations are shown in red text; deletions are shown with red dashes (−). The sequence numbering is provided relative to each ASLV Env protein.

**Figure 9:**
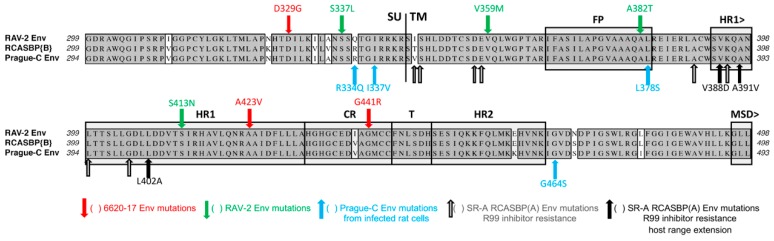
A summary and comparison of the ASLV c-terminal SU and N-terminal TM glycoprotein wild type and escape mutations identified using various genetic-based selections strategies limiting the entry and subsequent production of replication-competent ASLVs. Comparison of the C-terminal end of SU and N-terminal end of TM glycoproteins of RAV-2, RCASBP(B)SR-A, and Prague C. The sequence alignments were done using the ClustalW program in MacVector 14.5.3: identical residues are shaded; conserved residue differences are in boxes, and nonconserved residue differences are unmarked.

The major studies that used genetic selection strategies to evolve ASLV entry variants are each summarized below. Three types of ASLV envelope glycoprotein variants were selected. One type of acquired mutations that cluster mainly in the hr1 and vr3 hypervariable regions of the SU glycoproteins (summarized in Figure 8), and resulted in the loss of receptor specificity leading to expansion of receptor usage: changes in the 2–3 ASLV entry mechanism step of receptor priming/initial trigger of conformation changes (Figure 6). A second type of acquired mutations occurred in the C-terminal region of SU and/or the extracellular region of TM glycoproteins (summarized in Figure 9), altering the two-step envelope glycoprotein fusion mechanism to circumvent a normal requirement often resulting in a partially activated form of glycoprotein trimer more easily induced to facilitate entry (Figure 6). And finally, there were examples of a combination of both types of mutations needed to overcome a particular evolutionary hurdle.

The Schmidt–Ruppin Subgroup A envelope glycoproteins in RCASBP(A) evolve to evade the antiviral effects of quail Tva receptor immunoadhesin. We had shown that receptor immunoadhesins of both the chicken and quail Tva receptor homologs when delivered by an RCAS vector significantly blocked ASLV(A) virus entry both in cultured chicken cells with a >200-fold antiviral effect, and protected >98% birds from subsequent ASLV(A) challenge [60]. A chicken DF-1 cell line was developed that expressed high levels of the quail sTva-mIgG immunoadhesin that could inhibit infection by ASLV(A) by >15,000-fold. These cells were then challenged with different amounts of RCASBP(A) in an effort to select virus variants that could evade the antiviral effect of the quail sTva-mIgG inhibitor. Three viral variants were selected with mutations in the hr1 hypervariable region of the SU glycoprotein that reduced the binding affinity to the quail sTva-mIgG: Y142N (324-fold), E149K (32-fold), and the Y142N/E149K double mutant (4739-fold) [22]. Unexpectantly, based on our understanding of the Tva receptor at the time, all three mutant glycoproteins retained wild type levels of binding affinity for chicken sTva-mIgG. All three variants infect cells with a chicken Tva receptor at wild type levels but were 10-fold (Y142N), 2-fold (E149K), and 600-fold (Y142N/E149K) less efficient infecting cells expressing the quail Tva receptor. As discussed earlier, these variant glycoproteins that now preferred the chicken Tva receptor over the quail Tva receptor allowed the fine mapping of new determinants in the chicken versus the quail Tva receptor homologs critical for efficient interaction with ASLV(A) glycoproteins and virus entry. Upon analysis with receptor interference assays, these selected glycoprotein mutations may also broaden receptor usage but only when in the presence of the quail sTva-mIgG inhibitor.

A repetition of the same experiment, RCASBP(A) challenge of the same chicken DF-1 cell line expressing high levels of quail sTva-mIgG, selected an altered population of escape variants [23]. In this experiment, 80% of the escape population expressed the same hr1 mutation Y142N as the first experiment. However, the other 20% of the population contained two variants, both with one mutation in hr1 and one mutation in vr3: one variant contained the W141G/K261E mutations while the other variant W145R/K261E mutations. The two new viral variants escape primarily by lowering the binding affinity of the mutant glycoproteins for quail sTva-mIgG immunoadhesin inhibitor while retaining wild type binding affinity for the chicken Tva receptor. However, a secondary phenotype of the new variants was an alteration in the receptor interference patterns compared to wild type ASLV(A), indicating a possible interaction of these variant glycoproteins with other cellular receptors including Tvb and Tvc. Another indication of altered receptor usage was the replication of the W141G/K261E variant caused a transient period of cytotoxicity in DF-1 cells.

The Prague Subgroup B envelope glycoproteins in tdPrB-RSV evolve to extend receptor usage in a mixture of susceptible and resistant avian cells. The chicken Tvb^S1^ receptor supports the infection of subgroup B and D ASLVs while the quail Tvb^Q^ receptor only supports subgroup E ASLV entry. To test the hypothesis that the ASLVs altered receptor usage in response to the selective pressures of receptor polymorphisms in normal hosts, a subgroup B Prague B RSV (PrB) was passaged in a mixture of chicken CEFs expressing the Tvb^S1^ receptor and resistant to subgroup E, and quail cells expressing the Tvb^Q^ receptor and resistant to subgroups B and D. A variant virus was selected with expanded receptor usage with two mutations in the hr1 region of the SU glycoprotein, L154S and T155I, and to Tvb receptors that support B and D, and now E, subgroup viruses [70]. As determined later, only the L154S mutation was necessary to extend the receptor usage, and this mutation also extended the host range to nonavian cell types [71]. Receptor interference studies demonstrated that the PrB/L154S mutant virus efficiently interacted with both the Tvb^S1^ and Tvb^Q^ receptors, but the mutant has another means of infection not dependent on these receptors. The PrB/L154S mutant virus also induced a transient cytotoxicity when replicating in DF-1 cells but not CEFs compared to no cytotoxicity observed with the wild type PrB virus replication. Again, as observed in other studies, the acquisition of mutations that expand ASLV receptor usage to overcome a selective bottleneck inhibiting virus entry often results in a more cytotoxic virus [72].

The Schmidt–Ruppin Subgroup A envelope glycoproteins in RCASBP(A) evolve to evade the antiviral effects of the SR-A glycoprotein immunoadhesin. A DF-1 cell line was generated that expressed high levels of the subgroup A SU immunoadhesin, SUA-rIgG, to specifically bind/block the Tva receptor and significantly reduce infection by ASLV(A) viruses. Subsequent challenge of this cell line with RCASBP(A), after a significant delay, selected a variant virus resistant to the SUA-rIgG receptor interference [45]. The variant had acquired a six-amino acid deletion in the hr1 domain of the SUA glycoprotein, residues 155–160, that expanded its ability to use other receptors for entry while maintaining the ability to use the Tva receptor. This was demonstrated using receptor interference assays that showed the Del155-160 virus could now infect DF-1/RCAS(A) infected cells >100-fold more efficiently while DF-1/RCAS(B) and DF-1/RCAS(C) cells blocked infection by 5–10-fold compared to wild type RCASBP(A) indicating the variant was now able to use the Tvb and Tvc receptors for entry (Figure 6A). This expansion of receptor usage by the Del155–160 virus came with a cost to replication as indicated by the maximum titers produced were ~10^5^ ifu/mL, a similar titer to RCASBP(B) and RCASBP(C) viruses, and likely due to the observed transient period of cytotoxicity induced by the Del155-160 virus replicating in DF-1 cells.

The Schmidt–Ruppin Subgroup A mutant envelope glycoprotein RCASBP(A)Del155–160 that evolved to evade the antiviral effects of the SR-A glycoprotein immunoadhesin, could evolve further to evade the antiviral effects of quail Tva receptor immunoadhesin. The evolution of the subgroup A to E ASLVs likely occurred by an accumulation of a series mutations reflecting the exposure of the evolving virus to different blocks to infection over time. To add a second evolutionary step, we used the selected Del155–160 virus to challenge the DF-1 cell line expressing high levels of quail sTva-mIgG immunoadhesin [63]. Since the Del155–160 virus still binds Tva at wild type levels, sTva-mIgG exposure significantly inhibited infection of these cells. In one experiment, a mixture of three different resistant variants was selected after a long delay with one or two mutations in the SU glycoprotein hr1 variable region in addition to the Del155–160: G133D, G133D+L143P, and G133D+Y142H. Only the two double mutation variants were resistant to quail sTva-mIgG inhibition. A second experiment selected a single resistant variant with a mutation in the SU vr3 region in addition to the Del155-160; G268E. All of the mutations in the SU glycoprotein significantly reduce the binding affinity for the Tva receptor. The receptor interference patterns of all four selected variants were further altered compared to the original Del155–160 virus with a significantly reduced ability to infect all three DF-1/A, DF-1/B and DF-1/C infected cell cultures (Figure 6A). This expansion of receptor usage, loss of any receptor specificity, also resulted in an addition loss of titer with the selected variant viruses only reach titers of ~10^4^ ifu/mL.

The RCASBP(C) envelope glycoprotein consisting of the Prague Subgroup C SU hypervariable region fused to the rest of the SR-A envelope glycoprotein could evolve to infect Tvc receptor negative chicken cells. Chicken Line 15I5 does not express a functional Tvc receptor. Line 15I5 CEFs were challenged with RCASBP(C) which expresses the Prague C SU variable region fused to the SR-A SU C-terminal and TM regions. A variant virus pool was selected with mutations in the SU hr1 variable region only after using a large challenge dose and a significant replication delay: all variants acquire a 20-amino acid deletion, 144–160, and 20% also have a F142S mutation [63]. Only the Del144–160+F142S variant could replicate well in Line 15I5 CEFs. The mutations significantly reduced the binding affinity for the Tvc receptor and the receptor interference pattern is altered compared to wild type RCASBP(C) with significantly reduced abilities to infect DF-1/A (10-fold) and DF-1/B (100-fold) infected cells while still significantly blocked infecting DF-1/C infected cells. Once again, the cost of expanded receptor usage results in replication to a 10-fold lower titer compared to wild type: ~10^4^ ifu/mL compared to 10^5^ ifu/mL RCASBP(C).

The RCASBP(B) envelope glycoprotein consisting of the RAV-2 Subgroup B SU hypervariable region fused to the rest of the SR-A envelope glycoprotein could evolve to evade the antiviral effects of the Tvb^S3^ immunoadhesin. RCASBP(B) expresses a combination of the subgroup B SU variable region of RAV-2 fused to the SR-A SU C-terminal and TM regions. A DF-1 cell line was generated that expresses high levels of the sTvb^S3^-mIgG immunoadhesin that binds to the subgroup B ASLV glycoproteins blocking access to cellular Tvb receptors and infection. The DF-1/sTvb^S3^-mIgG cell line was challenged with RCASBP(B) or RCASBP(RAV2) that contained the entire wild type RAV-2 *env* gene [64]. A variant resistant to the antiviral effects of the sTvb^S3^-mIgG immunoadhesin was only selected from the RCASBP(B) challenged culture; RCASBP(RAV2) failed to evolve a resistant variant even after multiple attempts. All selected variants contained a 7-amino acid deletion in the SU hr1 region of the RCASBP(B) SU glycoprotein, residues 136–142. As expected, the Del136-142 knocked out the ability of the RCASBP(B)/Del136-142 glycoproteins to bind the sTvb^S3^-mIgG immunoadhesin. There are only 8-amino acid differences between the extracellular C-terminal SU and TM regions of RAV-2 and SR-A (Figure 9).

The RCASBP(RAV2) envelope glycoprotein consisting of the entire RAV-2 Subgroup B envelope glycoprotein could not evade the antiviral effects of the Tvb^S3^ immunoadhesin. We constructed the same Del136-142 deletion into the RCASBP(RAV2) *env* gene to test whether this mutation would rescue the ability of RAV-2 to replicate in the presence of sTvb^S3^-mIgG [64]. Unexpectantly, not only did the Del136-142 deletion not rescue RCASBP(RAV2)/Del136-142 replication on the sTvb^S3^-mIgG selective cells until additional mutations were acquired, but the production of detectable RCASBP(RAV2)/Del136-142 virus on DF-1 cells was significantly delayed and also needed the acquisition of addition mutations. Variants were selected from the RCASBP(RAV2)/Del136-142 culture with two different mutations in the TM region of RAV-2 glycoprotein in RCASBP(RAV2); V359M and A382T. The major variant selected from RCASBP(RAV2)/Del136-142 replication in the presence of sTvb^S3^-mIgG contained four mutations in the C-terminal SU and TM regions of Rav-2 glycoprotein: S337L, V359M, A382T, and S413N. We constructed several combinations of these mutations but resistance to sTvb^S3^-mIgG required at least the three mutations in TM (RAV-2/Del+VAS) or all four mutations (RAV-2/Del+SVAS). These mutations altered the pattern of receptor interference of these variant viruses somewhat compared to wild type RAV-2 or RCASBP(B) (Figure 6B), but significantly extended the host range of the variants for infection of several mammalian cells compared to wild type RAV-2 (Figure 6C). RAV-2 required mutations in both the SU and TM glycoproteins to evolve a variant that could plicate well and resist the inhibition of the sTvb^S3^-mIgG immunoadhesin.

Recombination between RCASBP(A) and RAV-2 envelope glycoproteins provided an advantage for replication in the presence of the Tvb^S3^ immunoadhesin. RCASBP(A) was used to deliver the sTvb^S3^-mIgG immunoadhesin gene in chicken embryos to express the immunoadhesin in virtually all cells and tissues of the hatched chicks [64]. The chicks expressing sTvb^S3^-mIgG were significantly and specifically resistant to a challenge with subgroup B wild type RAV-2 virus but not subgroup C RAV-49 virus: 15 of 17 birds (88%) challenged with RAV-2 did not produce detectable subgroup B ASLV; 100% of birds challenged with RAV-49 produced subgroup C ASLV. The subgroup B ALV *env* gene populations in the two positive RAV-2 challenged birds were cloned and sequenced to determine if a resistant virus was selected. One of the birds, 6590, only contained wild type RAV-2 *env* genes and expressed the lowest level of sTvb^S3^-mIgG likely indicating incomplete delivery and expression of the immunoadhesin throughout the bird allowing some subgroup B virus replication. The second bird, 6620, contained a mixture of RAV-2 and RAV-2/SR-A recombinant *env* genes from recombination between the RCASBP(A) and RAV-2 *env* genes. Interestingly, RAV-2 acquired much of the C-terminal SU and N-terminal TM region of the SR-A *env* gene, a construct very similar to the RCASBP(B) vector with RAV-2 SU variable regions fused to the rest of the SR-A *env* gene. We further characterized several molecular clones including clone 6620-17, that contained the recombination plus additional mutations in the SU glycoprotein: R196K and Y201N in the region between hr1 and hr2; and a 27 bp duplication encoding a 9-amino acid duplication in the RAV-2 hr2 variable region. However, none of the representative variant viruses with *env* genes found in bird 6620, including 6620-17, which were resistant to sTvb^S3^-mIgG inhibition.

To determine what additional mutations were needed for the 6620-17 virus to acquire resistance to the sTvb^S3^-mIgG immunoadhesin, the virus was passaged in the DF-1 cell line expressing high levels of sTvb^S3^-mIgG. After a significant delay, variant viruses were selected with mutations in the vr3 variable region of SU and in the hinge region of TM between the HR1 and HR2 regions. Both mutations were required for replication in the presence of sTvb^S3^-mIgG, however, other TM mutations could rescue a 6620-17/E266K alone mutant: A423V; A423V+G441E; and the loss of the E266K mutation but with the addition of the A423V+G441E mutations.

### The Cell Entry Characteristics of RCASBP(B) versus RAV-2 Wild Type Envelope Glycoproteins

The acquisition of escape mutations in non-SU glycoprotein hypervariable regions upon selective pressure on virus entry was surprising. Indeed, there are only eight amino acid differences between the RCASBP(B) and RCASBP(RAV2) viruses to account for the inability of RCASBP(RAV2) to escape sTvb^S3^-mIgG inhibition: five differences in the C-terminal region of SU (2 nonconserved changes) and three differences in TM (2 nonconserved changes). The analysis of the biochemical properties of the RCASBP(B) and RCASBP(RAV2) envelope glycoprotein trimers demonstrate fundamental differences between the two glycoproteins in their ability to execute virus entry with the two-step mechanism of ASLV fusion process [64]. The mature RCASBP(B) glycoproteins appear to be more stable compared to the RCASBP(RAV2) glycoproteins upon conformation change triggered by heat with significant levels of triggered RCASBP(RAV2) glycoproteins occurring at low temperatures. In addition, the RCASBP(RAV2) glycoproteins did not appear to require a subsequent exposure to low-pH after receptor induced conformational changes as would be expected and is observed with RCASBP(B) glycoproteins. Changing the four conserved amino acid differences converted the RCASBP(RAV2) glycoprotein properties to RCASBP(B) glycoproteins, and vice versa. This indicates that mutations altering the two-step entry fusion mechanism of ASLV provides another evolutionary escape mechanism perhaps independent of hypervariable mutations in the SU glycoprotein that alters receptor-glycoprotein interactions.

The Schmidt–Ruppin Subgroup A envelope glycoproteins in RCASBP(A) evolve to evade the antiviral effects of the R99 peptide fusion inhibitor. RCASBP(A) escape variants were selected for resistance to a peptide inhibitor (R99) that bound the HR2 region and prevented the formation of 6HB necessary for membrane fusion and virus entry. In addition to the expected mutations in HR1 that would compensate, several variants were isolated that had mutations in the N-terminal region of TM outside the HR1 region [69,73]. Finally, some of these mutations in the HR1 region of the TM glycoprotein not only escaped the R99 inhibition, but also expanded receptor tropism to nonavian cells: V388D, A391V, and L402A. These three mutations significantly lowered the normally strict requirement for physiological temperatures to trigger conformational changes in the SU glycoproteins of the trimer upon receptor binding perhaps expanding the use of cell surface proteins besides Tva that could trigger the initial step of entry.

The entire Prague Subgroup C envelope glycoprotein in the RCAS, RCASBP(PrC-RSV) could evolve to infect receptor-negative mammalian cells. Despite ASLVs only infecting avian species naturally, Jan Svoboda’s group had shown that ASLVs could be used to infect hamsters and rats experimentally with RSVs and used these infected transformed cells to study the RSV life cycle [74,75,76]. Recently, they investigated the mutations acquired by the Prague C RSV *env* gene that allowed the entry into rodent cells: first by infecting newly born rats with PrC-RSV infected chicken tissue and generating the XC-RSV cell line; followed by XC-RSV rescued from the rat cell line used to infect newly born hamsters and generating the H20-RSV hamster cell line [77]. Eight amino acid substitutions were found in the H20-RSV *env* gene sequence: three mutations near the N-terminus of the SU glycoprotein, two mutations in the C-terminus of SU, and three mutations in the TM glycoprotein. Unexpectantly, there were no mutations in the SU glycoprotein hr1, hr2, or vr3 hypervariable regions critical for receptor specificity and host range. Upon analysis, the key mutations that allowed the expanded host range were identified as the mutations in the TM glycoprotein, especially the L378S mutation in the fusion peptide. Biochemical characterization determined that the L378S mutation altered the H20-RSV envelope glycoprotein trimers to no longer require a receptor to prime the initial triggering of the SU conformational changes: the H20-RSV glycoprotein trimer could be induced to form TM oligomers most efficiently with low pH exposure alone even at room temperature. The ability to infect mammalian cells was transferred to a subgroup B ALV by constructing a RCASBP(B) vector but with the H20-RSV TM glycoprotein. Thus, they concluded the H20-RSV virus was able to extend its host range to mammalian cells due to the pre-activation of the mutant glycoproteins and not to the use of a new mammalian cell surface protein as a receptor.

## 7. Summary/Reprise

The ASLVs continue to provide a powerful experimental system for studying the mechanisms involved in enveloped virus entry and the evolution of receptor usage and host range expansion. The highly related subgroup A-E ASLVs envelope glycoproteins provide an example of the evolution to use alternate host cell surface proteins as receptors that have been identified and cloned. The unusual two-step entry fusion process of ASLV allows a detailed biochemical dissection of the entry steps not possible with other enveloped viruses. Replication-competent ASLV viruses, and the RCAS series of vectors based on them, have enabled the use of genetic selection strategies to pressure the virus to evolve the envelope glycoproteins to alter receptor binding affinity, receptor usage including mutations that expand their host range. In this way, the virus has naturally identified multiple residues/regions of the envelope glycoprotein important for productive viral glycoprotein receptor interactions as well as provided insights into the subsequent conformation changes in the trimer required for the fusion of the viral and cellular membranes.

The reverse engineering studies summarized above sought to experimentally force the subgroup A to E ASLV envelope glycoproteins to evolve in order to efficient infect cells possibly mimicking the evolutionary process that resulted in this highly homologous group of glycoproteins but with exquisite receptor usage specificity. These studies highlight the important interplay of SU and TM glycoproteins in the active, metastable trimer necessary to promote efficient ASLV entry. The results identify several themes in mutations that evolved that altered Env glycoprotein receptor binding affinity and/or specificity.

1. Small 1–2-amino acid changes in the hr1/vr3 regions can alter receptor binding affinity and receptor usage to even preferentially exploit the subtle differences that exist between one receptor homolog and another, e.g., chicken Tva versus quail Tva; expansion to use chicken Tvb^S1^ and quail Tvb^Q^. In these cases, wild type binding affinity was retained for one receptor homolog while significantly reducing the binding affinity to the other homolog, while maintaining wild type levels of viral replication and titer.
2. Often, the initial evolutionary step is a deletion mutation in the hr1 C5–C6 loop of the SU glycoprotein that knocks out the normal receptor binding affinity and broadens receptor usage to other cell surface proteins including other known ASLV receptors, and may or may not also broaden host range.
3. Subtle differences in the amino acid sequence in the C-terminal region of the SU glycoprotein and/or the extracellular region of the TM glycoprotein can affect the specificity and efficiency of the two-step ASLV membrane fusion process. The mutations that alter the biochemical properties of the fusion process often result in an “activated” envelope glycoprotein trimer that can more easily, and with less specificity, facilitate membrane fusion and virus entry.
4. In general, expansion of ASLV receptor usage away from the extreme one receptor specificity leads to a loss of viral replication fitness and lower maximum titers likely due to the glycoprotein variants causing cytotoxic effects in the cells. While the ability of a virus to use multiple different receptors would seem to be an evolutionary advantage, these studies observe a deleterious effect of an expanded receptor usage that would likely pressure for the glycoprotein to acquire additional mutations to overcome this disadvantage presumably increasing receptor specificity.

While the ASLV experimental system has been extremely useful to study functional aspects of enveloped virus entry, we still cannot put these functional determinants into a structural framework since there are no atomic level structures of the ASLV glycoproteins. Perhaps with the further development of cryoelectron tomography, useful structures of the ASLV envelope glycoprotein trimers on virions will soon be generated.

**Figure 10:**
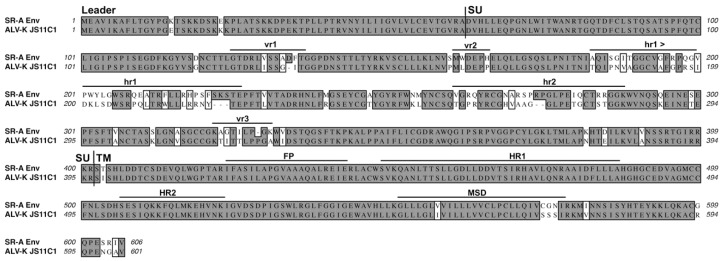
A comparison of the SR-A subgroup A and the JS11C1 subgroup K envelope glycoproteins. See Figure 2 legend for analysis details. JS11C1, Genbank #KF46200.1.

Recently, a new group of related ASLV viruses has emerged in chickens that have tentatively been grouped into a new envelope glycoprotein subgroup, K, based on sequence analysis [78]. The ASLV-K isolate sequences cluster separately from the subgroup A to E ASLVs. However, the envelope glycoproteins of a prototypic ASLV-K isolate, JS11C1, are 86% identical to the SR-A subgroup A glycoproteins with the majority of the amino acid variation occurring in the SU glycoprotein hypervariable regions (Figure 10). It is interesting to note that there are several amino acid differences in the C-terminal region of SU that are similar to those in RAV-2 where they altered the fusion process (Figure 9). Dr. Jiri Hejnar at the Czech Academy of Sciences is reporting that the JS11C1 isolate, and most likely all of the subgroup K ASLVs, use Tva as their receptor [79]. It remains to be determined how the ASLV-K glycoproteins interact with Tva and whether the interactions map to different determinants than those used by ASLV-A glycoproteins.

## Figures and Tables

**Table 1:** The characterized chicken ASLV receptor alleles including the genetic defects and phenotypes of the known ASLV receptor resistance alleles.

Allele	Chicken Line	Mutation	Phenotype	Ref.
***tva^s^***	Line H6 Line 0	Wild type	Tva receptor conferring susceptibility to ASLV(A) infection.	[57]
***tva^r^***	Line C Line Rh-C	Single nucleotide mutation in *tva^s^* resulting in a Cys40Trp substitution.	Structural change in Tva due to alteration of the disulfide bond pattern. Drastically lowered binding affinity for ASLV(A) envelope glycoproteins.	
***tva^r2^***	Line 7_2_	Four-nucleotide insertion in *tva^s^* changing reading frame.	Predicted to lead to a complete absence of the Tva protein.	
***tvb^s1^***	Line 15_B_1	Wild type	Tvb^S1^ receptor conferring susceptibility to ASLV subgroups B, D, and E.	[49,58]
***tvb^s3^***	Line 0	Single nucleotide mutation in *tvb^s1^* resulting in a Cys62Ser substitution.	Structural change in Tvb^S1^ due to alteration of the disulfide bond pattern. Drastically lower binding affinity for only ASLV(E) envelope glycoproteins.	[49,58]
***tvb^r^***	Line 7_2_	Single nucleotide mutation in *tvb^s1^* resulting in a premature stop codon.	Predicted to lead to the production of a severely truncated protein at amino acid 57 of the Tvb^S1^ protein.	[58]
***tvb^r2^***	Line M	Single nucleotide mutation in *tvb^s1^* resulting in a Cys125Ser substitution.	Structural change in Tvb^S1^ due to alteration of the disulfide bond pattern. Lowered binding affinity for ASLV(B) and ASLV(D), and drastically lower binding affinity for ASLV(E) glycoproteins.	[52]
***tvc^s^***	Line H6	Wild type	Tvc receptor conferring susceptibility to ASLV(C) infection.	[54]
***tvc^r^***	Line 15 Line 15I5	Single nucleotide mutation in *tvc^s^* resulting in a premature stop codon.	Predicted to lead to the production of a severely truncated protein at amino acid 55 of the Tvc protein.	
